# Eugenol Reduced ΜPO, CD45 and HMGB1 Expression and Attenuated the Expression of Leukocyte Infiltration Markers in the Intestinal Tissue in Biliopancreatic Duct Ligation-Induced Pancreatitis in Rats

**DOI:** 10.3390/medicina60010074

**Published:** 2023-12-30

**Authors:** Panagoula Oikonomou, Christina Nikolaou, Fotini Papachristou, Apostolos Sovatzidis, Maria Lambropoulou, Charikleia Giouleka, Vasileios Kontaxis, Dimitrios Linardoutsos, Apostolos Papalois, Michael Pitiakoudis, Alexandra Tsaroucha

**Affiliations:** 1 Postgraduate Program in Hepatobiliary and Pancreatic Surgery, 2nd Department of Surgery, Faculty of Medicine, Democritus University of Thrace, 68100 Alexandroupolis, Greece; t.sovatzidis@gmail.com (A.S.); charagkrx@gmail.com (C.G.); kontaxisv@gmail.com (V.K.); dlinardoutsos@hotmail.com (D.L.); mpitiak@med.duth.gr (M.P.); atsarouc@med.duth.gr (A.T.); 2 Laboratory of Experimental Surgery and Surgical Research, Faculty of Medicine, Democritus University of Thrace, 68100 Alexandroupolis, Greece; cnikolaou3@gmail.com (C.N.); fpapachr@med.duth.gr (F.P.); 3 Laboratory of Histology-Embryology, Faculty of Medicine, Democritus University of Thrace, 68100 Alexandroupolis, Greece; mlambro@med.duth.gr; 4 Experimental Research Center, ELPEN Pharmaceuticals, Pikermi, 19009 Athens, Greece; apostolospapalois@gmail.com

**Keywords:** acute pancreatitis, IL-6, TNFα, MPO, HMGB1, CD45

## Abstract

*Background and Objectives*: Inflammation and dysregulation in the intestinal barrier function in acute pancreatitis (AP) trigger pancreatic lesions, systemic inflammatory response, and multiple organ dysfunction. Eugenol, as the main component of clove (Syzygium aromaticum), is known for its antioxidant and anti-inflammatory properties. We studied the potentially beneficial effect of eugenol in a rodent model of biliopancreatic duct ligation-induced AP. *Materials and Methods*: Rats were randomly divided into three groups: Sham, AP, and AP + eugenol (15 mg/kg/day). Serum TNFα, IL-6, IL-18, and resistin levels, as well as IL-6, TNFα, MPO, HMGB1, and CD45 tissue expression, were determined at various timepoints after the induction of AP. *Results*: Eugenol attenuated hyperemia and inflammatory cell infiltration in the intestinal mucosal, submucosal, and muscular layers. IL-6 and resistin serum levels were significantly reduced in the AP + eugenol group, while serum TNFα and IL-18 levels remained unaffected overall. TNFα pancreatic and intestinal expression was attenuated by eugenol at 72 h, while IL-6 expression was affected only in the pancreas. MPO, CD45, and HMGB1 intestinal expression was significantly reduced in eugenol-treated rats. *Conclusions*: Eugenol managed to attenuate the inflammatory response in the intestine in duct ligation-induced AP in rats.

## 1. Introduction

Acute pancreatitis (AP) is an inflammatory disease of the exocrine pancreas, characterized by various symptoms of different severity [1,2]. Following the initiation of the inflammatory response by damage-associated molecular patterns (DAMPs), a subsequent event that plays a crucial role in the pathogenesis of AP is the change in the integrity of the intestinal barrier function. Changes in the permeability of the bowel result in bacterial translocation to the pancreas and other organs, which eventually leads to pancreatic necrosis, systemic inflammatory response syndrome, and multiple organ dysfunction syndrome [3,4].

Oxidative stress and inflammation seem to play a significant role in the pathogenesis of the disease [5,6,7,8]. The upregulation of cytokines IL-6, TNFα, IL-1β, IL-18, and IL-10 in the pancreas takes place from the early stages of AP and seems to trigger the succeeding inflammatory response [9,10,11,12,13]. The adipokine resistin, apart from its significance in obesity and the development of type 2 diabetes, is also implicated in inflammation and oxidative stress in general [14,15,16,17,18]. It is expressed in adipose tissue, pancreatic islets, adrenal glands, skeletal muscle, and the gastrointestinal tract in rats [19,20]. Serum resistin levels were significantly increased in AP patients [21], and they were positively correlated with serum levels of C-reactive protein, TNFα, and IL-1β, as well as pancreatic lesions, in cerulein- and L-arginine-induced AP in rats [22]. However, its value as a serum marker for predicting AP severity is unclear [23,24]. CD45 is a transmembrane glycoprotein implicated in immune response regulation via antigen-receptor signaling [25,26]. Apart from nucleated hematopoietic cells, it is also expressed by pancreatic acinar cells [27,28]. De Dios et al. reported the translational and later transcriptional regulation of CD45 expression in acinar cells at the early stages of duct ligation-induced AP in rats. Their results suggested the downregulation of CD45 expression by ROS generation during AP and a negative correlation between CD45 and cytokine production in pancreatic acinar cells via the inhibition of MAPK dephosphorylation by CD45 [29]. Myeloperoxidase (MPO) belongs to heme peroxidases and is mainly expressed by neutrophils. It is released into the phagolysosomal compartments or the extracellular space in the early phase of an immune response. MPO activation leads to the generation of hypohalous acids that serve as body defense mechanisms against bacterial infections [30,31]. Serum MPO levels were positively correlated with cytokine serum levels and the severity of AP [32]. According to reports, experimentally induced AP in rodents induced MPO activity in the pancreas, the lung, and the large intestine [33,34]. L-arginine-induced AP in rats significantly reduced CD45 expression in acini and isolated acinar cells, damaged the function of the intestinal mucosal barrier, and increased TNFα serum levels and pancreatic MPO activity [35]. The extracellular release of HMGB1, a DAMP molecule, under stress conditions has been associated with the pathogenesis of various diseases [36,37,38]. In AP, HMGB1 is actively released by damaged cells, while in severe necrotizing AP, it is also passively released by necrotic cells of the pancreas and other organs. Therefore, it has been suggested that it acts as a proinflammatory late cytokine and contributes to further tissue injury and organ failure [39,40]. Elevated HMGB1 serum levels were positively correlated with lactate dehydrogenase, C-reactive protein, total bilirubin, and disease severity in severe AP patients [41], while HMGB1 expression in the ileum was increased in an experimental model of severe AP in rats [42].

Eugenol, a natural phenolic compound, is the main component of Syzygium aromaticum essential oil [43,44]. It possesses anti-inflammatory, antinociceptive, antimicrobial, gastroprotective, and anti-oxidant properties [45,46,47,48,49,50,51,52]. We previously reported the protective effect of eugenol on the pancreas and kidneys in duct ligation-induced AP in rats [53]. Eugenol also exhibited a protective effect on the pancreas and lungs in severe necrotizing AP in rats [54]. It also reduced the inflammatory response and preserved the intestinal barrier function in an in vitro lipopolysaccharide-induced inflammation model [55].

In the present study, we investigated the anti-inflammatory effect of eugenol in the intestine in an experimental model of duct ligation-induced AP. More specifically, we determined serum TNFα, IL-6, IL-18, and resistin levels as well as IL-6, TNFα, MPO, HMGB1, and CD45 tissue expression.

## 2. Materials and Methods

### 2.1. Animal Care and Handling

The present animal study was licensed by the Official Veterinary Authorities of the Prefecture of Attica according to the requirements set by the national legislation on the use of animals for scientific purposes. Experimental procedures and animal handling conformed to the National Research Council Guide for the Care and Use of Laboratory Animals and the Directives 2010/63/EU of the European Union and 86/609/EEC of the European Communities Council. The animals were obtained from the Pasteur Hellenic Institute (Athens, Greece), while all experimental surgical procedures were performed at the Experimental Research Center of ELPEN Pharmaceutical Co., Inc. (Pikermi, Athens, Greece).

In the present study, 120 male albino Wistar rats (3–4 months of age, 250–350 g) were used. All animals were housed in polycarbonate cages under controlled environmental conditions (room temperature: 22–25 °C; humidity: 55–58%; 12 h light/12 h dark cycle), and they were provided with commercial food and tap water ad libitum.

### 2.2. Experimental Design

Animals were randomly assigned into three main groups (sham, AP, and AP + eugenol), and they were then further divided into five subgroups depending on the euthanasia timepoints (6, 12, 24, 48, and 72 h). In the sham group (n = 20, 4/subgroup), all animals underwent open–close laparotomy; in the AP group (n = 50, 10/subgroup), biliopancreatic duct ligation was performed in order to induce AP; and in the AP + eugenol group (n = 50, 10/subgroup), apart from initiating AP, all animals received a daily dose of 15 mg/kg eugenol per os [53,56]. Eugenol (E51791, Sigma-Aldrich, Merck KGaA, Darmstadt, Germany) was diluted in corn oil at a concentration of 1.5 mg/mL. Sham and control groups received a daily dose of corn oil.

The protocols we adopted to perform anesthesia and induce AP via duct ligation have been previously described [53,56]. Briefly, prior to intubation, the animals were initially placed in an isoflurane induction chamber. Euthanasia was performed at predetermined timepoints by administering 100 mg/mL ketamine and 20 mg/mL xylazine. Analgesia was maintained by 2 mL/kg of butorphanol (Dolorex; Intervet/Schering/Plough Animal Health, Boxmeer, The Netherlands). Tissue and blood samples were collected for further analysis.

### 2.3. Histopathological Examination and Immunohistochemical Staining

Pancreatic and intestinal tissue specimens were fixed in 10% formalin and embedded in paraffin. Four-micron (4 μm)-thick tissue sections were cut using a Leica RM2030 rotary microtome (Leica Microsystems, Wetzlar, Germany) and mounted onto slides. Deparaffinization was performed at 80 °C for 30 min and was followed by xylene incubation and rehydration in descending dilutions of ethanol. Immunostaining was performed using the biotin-streptavidin method (Super Sensitive One-step Polymer-HRP Detection System kit, QD 630-XAKE, Biogenex, Fremont, CA, USA) according to the manufacturer’s instructions. Tissue sections were incubated for 60 min with rabbit polyclonal HMGB1 (dilution 1:250, PA1-16926, Thermo Scientific, Waltham, MA, USA), mouse monoclonal CD45 (dilution 1:250, sc-53047, Santa Cruz, Heidelberg Germany), rabbit polyclonal IL-6 (dilution 1:500, ab6672, Abcam, Waltham, MA, USA), rabbit polyclonal TNFα (dilution 1:1000, PAB8016, Abnova, Taoyuan City, Taiwan), and rabbit polyclonal MPO (dilution 1:400, A0398, Dako, Glostrup, Denmark) antibodies. Positive and negative controls were also included. Bound antibody complex visualization was performed by incubating the tissue sections with diaminobenzidine (DAB) for 10 min and counterstaining with Mayer’s hematoxylin. Sections were evaluated using a Nikon Eclipse 50i microscope (Nikon Instruments Inc, New York, NY, USA).

Immunohistochemical and histopathological evaluation was semiquantitative, and it was performed in a blind fashion. The grading score was based on the proportion of positive cells after scanning the entire section. Staining was graded as negative (0) when ≤10% of cells were stained, as weak (1) when 10–30% of cells were stained, as moderate (2) when 30–70% of cells were stained, and as strong (3) when >70% cells were stained. Histopathological examination of pancreatic and intestinal tissues was performed on hematoxylin/eosin-stained sections, while the severity of histological lesions was graded according to the following scoring system: 0, no lesions; 1, mild lesions; 2, moderate lesions; 3, severe lesions [57].

### 2.4. Determination of Serum Protein Levels

Serum IL-6 (ER2IL6, Thermo Scientific, Waltham, MA, USA), TNFα (RTA00, R&D Systems, Northampton, UK), IL-18 (orb50132, biorbyt, Cambridge, UK), and resistin (E02R0351, amsbio, Northampton, UK) levels were determined by ELISA according to the manufacturer’s instructions. Serum samples were diluted 1:2 for the determination of IL-18, and 1:10 for the determination of resistin protein levels. The optical density was measured at 450 nm. Serum total protein concentration was determined by the BCA protein assay (Thermo Scientific, Waltham, MA, USA). Measurements were expressed as pg or ng of the determined protein per mg of total protein.

### 2.5. Statistical Analysis

Statistical analysis was performed using the SPSS 19.0 statistical package (IBM, Armonk, NY, USA). All data were expressed as median and interquartile range. The Kolmogorov–Smirnov test was applied to analyze the distribution of the variables. The non-parametric Kruskal–Wallis and Mann–Whitney tests were used for comparisons among and within groups, respectively. Spearman’s Rank correlation coefficient (rho) was employed to identify positive or negative relationships among variables (strength of rho correlations: 0.00–2.00, negligible; 0.21–0.40, weak; 0.41–0.60, moderate; 0.61–0.80, strong; 0.81–1.00, very strong). All tests were two-tailed, and *p* values < 0.05 were considered statistically significant.

## 3. Results

### 3.1. Histopathological Findings

At 72 h, biliopancreatic duct ligation-induced AP elicited moderate hyperemia and mild infiltration of inflammatory cells in the intestinal mucosal, submucosal, and muscular layers (Table 1). These lesions were significantly reduced after eugenol treatment (*p* < 0.05). There were no significant differences in edema, mucosal atrophy, crypt epithelial cell hyperplasia, and necrosis among the three groups.

In the AP group, hyperemia was positively correlated with MPO (rho = 0.697, *p* < 0.001), CD45 (rho = 0.644, *p* < 0.001), HMGB1 (rho = 0.518, *p* < 0.001), and TNFα intestinal expression (rho = 0.722, *p* < 0.001), as well as IL-6 (rho = 0.585, *p* < 0.001) and TNFα pancreatic expression (rho = 0.789, *p* < 0.001). As expected, the recruitment of inflammatory cells into the intestine was positively correlated with hyperemia (inflammatory cell infiltration of the intestinal mucosa: rho = 0.651, *p* < 0.001; inflammatory cell infiltration of the intestinal submucosa: rho = 0.576, *p* < 0.001; inflammatory cell infiltration of the intestinal muscular layer: rho = 0.507, *p* < 0.001). Inflammatory cell infiltration of the intestinal mucosa and submucosa was positively correlated with MPO (mucosa: rho = 0.601; submucosa: rho = 0.688, *p* < 0.001), HMGB1 (mucosa: rho = 0.568; submucosa: rho = 0.597, *p* < 0.001), and TNFα intestinal expression (mucosa: rho = 0.788; submucosa: rho = 0.755, *p* < 0.001), as well as TNFα pancreatic expression (mucosa: rho = 0.594; submucosa: rho = 0.541, *p* < 0.001). Finally, inflammatory cell infiltration of the muscular layer of the intestinal tissue was positively correlated with MPO (rho = 0.515, *p* < 0.001), TNFα intestinal (rho = 0.606, *p* < 0.001) and pancreatic expression (rho = 0.510, *p* < 0.001), as well as IL-6 (rho = 0.544, *p* < 0.001) pancreatic expression.

In the AP + eugenol group, MPO was positively correlated with hyperemia (rho = 0.521, *p* < 0.001), as well as inflammatory cell infiltration of the intestinal mucosal (rho = 0.539, *p* < 0.001), submucosal (rho = 0.572, *p* < 0.001), and muscular layer (rho = 0.708, *p* < 0.001). Inflammatory cell infiltration of the intestinal mucosal and muscular layer was positively correlated with TNFα intestinal expression (mucosa: rho = 0.577; muscle layer: rho = 0.722, *p* < 0.001). The recruitment of inflammatory cells into the muscular layer of the intestinal tissue was also negatively correlated with secreted IL-6 (rho = −0.592, *p* < 0.001), while the recruitment of inflammatory cells in the intestinal submucosa was negatively correlated with HMGB1 (rho = −0.542, *p* < 0.001).

### 3.2. Eugenol Treatment Mainly Affects Serum IL-6 and Resistin Levels

**Figure 1:**
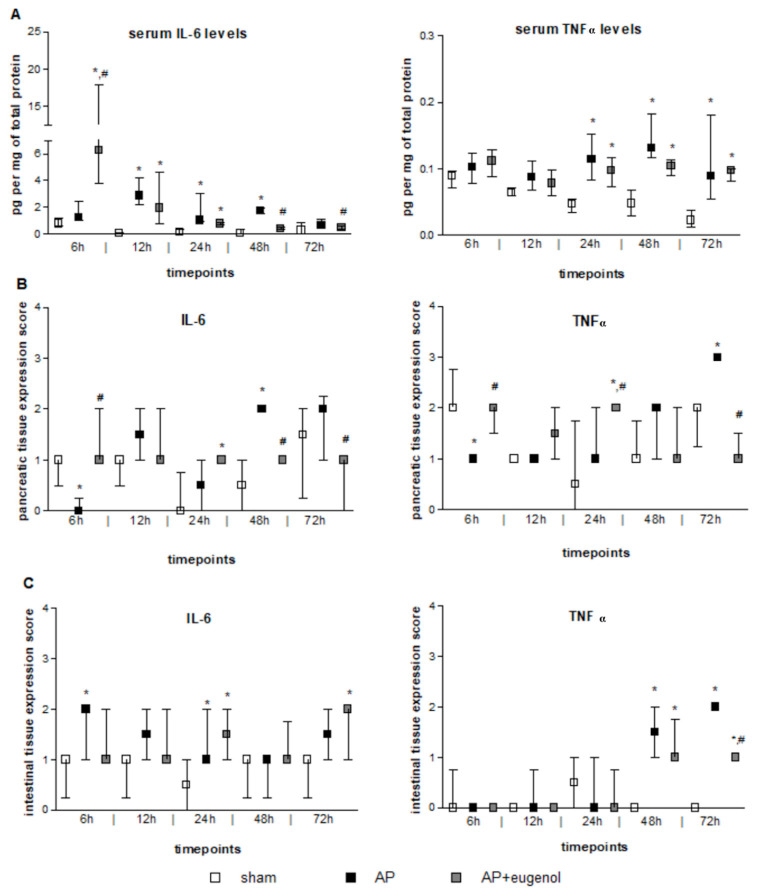
The effect of duct ligation-induced AP in eugenol-treated and untreated rats. (**A**) Serum IL-6 and TNFα levels, (**B**) IL-6 and TNFα pancreatic tissue expression, and (**C**) IL-6 and TNFα intestinal tissue expression. All data are presented as median and interquartile range. Where *, *p* < 0.05 vs. the sham group and where #, *p* < 0.05 vs. the AP group, at the corresponding timepoints.

According to our findings, serum IL-6 levels increased at 12 h after the induction of AP and remained elevated up to 48 h (*p* < 0.05, compared to the sham group) (Figure 1A). Eugenol treatment led to a very significant transient increase in serum IL-6 levels at 6 h, compared to both sham and AP groups (*p* < 0.01). This finding should be interpreted with caution due to the large variance within this particular subgroup. Furthermore, IL-6 levels significantly decreased at 48 h and 72 h compared to the AP group (*p* < 0.05). A time-dependent decrease in serum IL-6 was evident in the AP + eugenol group (*p* < 0.05).

Serum TNFα levels significantly increased after 24 h and up to 72 h in both AP and AP + eugenol groups compared to the sham group (*p* < 0.05) (Figure 1A). Eugenol treatment did not reduce serum TNFα levels compared to the AP group. There were no time-dependent changes in TNFα levels in any of the groups.

**Figure 2:**
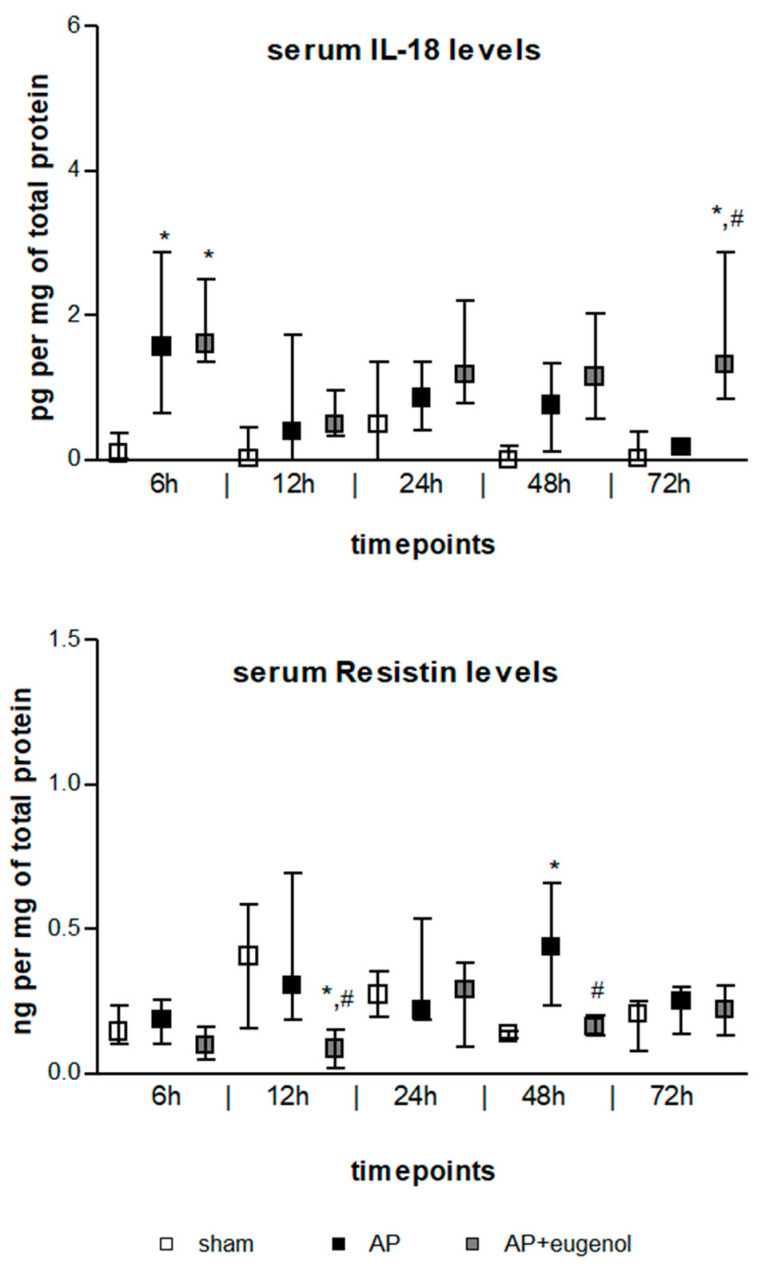
Serum IL-18 and resistin levels in duct ligation-induced pancreatitis in rats with or without eugenol treatment. All data are presented as median and interquartile range. Where * *p* < 0.05 vs. sham group and where # *p* < 0.05 vs. AP group, at the corresponding timepoints.

IL-18 serum levels exhibited a transient increase at 6 h in both AP and AP + eugenol groups compared to the sham group (*p* < 0.05) (Figure 2). Moreover, eugenol treatment led to a significant increase at 72 h (*p* < 0.05 vs. sham group, *p* < 0.01 vs. AP group). There were no time-dependent changes in IL-18 levels in any of the groups.

Resistin serum levels significantly increased at 48 h in duct ligation-induced AP compared to the sham group (*p* < 0.05) (Figure 2). The administration of eugenol reduced resistin levels in a statistically significant manner at 12 h and 48 h compared to the AP group (*p* < 0.05). Resistin significantly decreased at 12 h compared to the sham group (*p* < 0.05). There were no time-dependent changes in resistin levels in any of the groups.

### 3.3. Eugenol Affects IL-6 and TNFα Pancreatic Expression

IL-6 immunopositive cells were absent in the AP group at 6 h (*p* < 0.05 vs. sham and AP + eugenol groups) (Figure 1B). Overall, weak IL-6 immunostaining was noticed at all timepoints in the AP + eugenol group. Moderate IL-6 immunostaining was evident in the pancreatic tissue at 48 h in the AP group, which significantly differed from the other two groups (*p* < 0.05). The presence of eugenol significantly reduced IL-6 immunopositive cells at 72 h as well, compared to the AP group (*p* < 0.01).

TNFα immunopositive cells at 6 h were significantly fewer in the AP group compared with the sham and AP + eugenol groups (Figure 1B). Moderate TNFα immunostaining was evident in the AP + eugenol group at 6 h and 24 h (6 h: *p* < 0.01 vs. AP group; 24 h: *p* < 0.05 vs. sham and AP groups). The pancreatic tissue exhibited strong TNFα immunostaining at 72 h after the induction of AP, which significantly decreased following eugenol treatment (*p* < 0.001).

### 3.4. Eugenol Affects TNFα, MPO, CD45, and HMGB1 Expression in the Intestinal Tissue

IL-6 immunostaining was, in general, weak to moderate in the intestinal tissue in all groups (Figure 1C). A significant increase was evident at 6 h and 24 h in the AP group, and at 72 h in the AP + eugenol group, compared to the sham group (*p* < 0.05).

TNFα intestinal expression was absent in all groups up to 24 h (Figure 1C). At 48 h, weak to moderate immunostaining was evident in the AP and AP + eugenol groups (*p* < 0.05, compared to the sham group). At 72 h, TNFα immunostaining was moderate in the AP group, while it significantly differed compared to the AP + eugenol group (*p* < 0.01).

**Figure 3:**
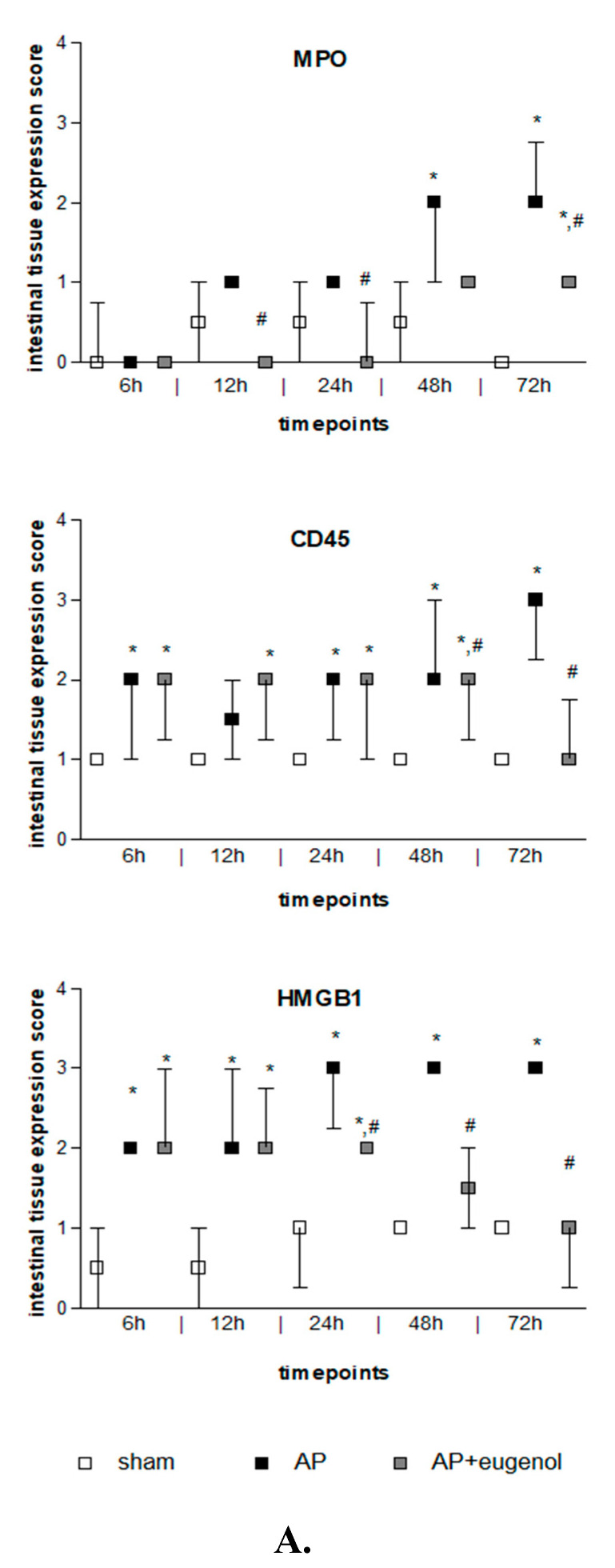

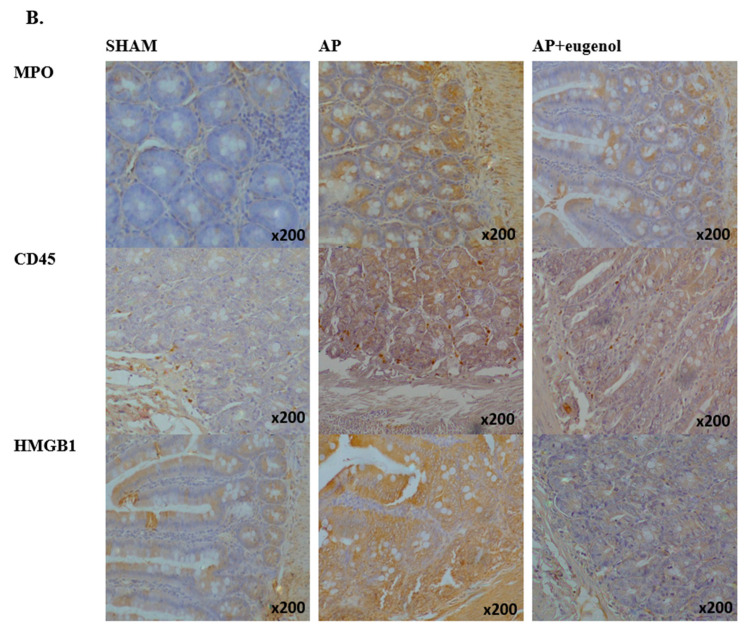
(**A**) MPO, CD45, and HMGB1 intestinal tissue expression. All data are presented as median and interquartile range. Where * *p* < 0.05 vs. the sham group, and where # *p* < 0.05 vs. the AP group, at the corresponding timepoints. (**B**) Immunohistochemical detection of MPO, CD45, and HMGB1 intestinal expression at 72 h in a rat model of biliopancreatic duct ligation-induced pancreatitis with or without eugenol treatment.

Duct ligation-induced AP increased MPO expression in the intestinal tissue at 48 h and 72 h compared to the sham group (*p* < 0.05). MPO was mainly detected in the epithelial cells of the intestinal mucosa and also in neutrophils and lymphocytes. Eugenol treatment following AP induction significantly reduced MPO immunopositive cells at 12, 24, and 72 h (*p* < 0.05 vs. AP group) (Figure 3). The presence of neutrophils and lymphocytes was limited at 72 h in the AP + eugenol group compared to the AP group.

CD45 expression was weak in the intestinal tissue in the sham group, while it generally increased after AP induction with or without eugenol treatment (*p* < 0.05 vs. sham group) (Figure 3). CD45 expression was upregulated in monocytes (macrophages, lymphocytes). Interestingly, it was detected in epithelial cells of the intestinal mucosa as well. CD45 expression significantly increased at 48 h compared to 6 h and 12 h in the AP group (*p* < 0.05) and remained at similar levels up to 72 h. Nevertheless, a time-dependent effect was not evident. Eugenol treatment following biliopancreatic duct ligation-induced AP effectively reduced the expression of CD45 at 48 h and 72 h compared to the AP group (*p* < 0.05) mainly by downregulating CD45 expression in monocytes.

Intestinal HMGB1 immunostaining was weak in the sham group and moderate to strong in the AP group at all timepoints tested (*p* < 0.01) (Figure 3). HMGB1 was detected in the cytoplasm of epithelial cells of the intestinal mucosa, where it displayed a punctuate staining, and rarely in cells of the interstitial connective tissue, in all groups. Moderate immunostaining was evident at 6 h and up to 24 h in the AP + eugenol group (*p* < 0.01 vs. sham group), while at 48 h, levels similar to the sham’s group expression levels were reached (*p* < 0.05, 24 h vs. 48 h and 72 h). Furthermore, eugenol treatment effectively reduced HMGB1 expression in the intestinal tissue at 24 h and up to 72 h compared to the AP group (*p* < 0.01).

In the AP group, MPO intestinal expression was positively correlated with IL-6 (rho = 0.686, *p* < 0.001) and TNFα (rho = 0.682, *p* < 0.001) pancreatic expression, as well as TNFα expression (rho = 0.684, *p* < 0.001) and CD45 (rho = 0.508, *p* < 0.001) and HMGB1 intestinal expression (rho = 0.540, *p* < 0.001). TNFα intestinal expression was positively correlated with IL-6 (rho = 0.585, *p* < 0.001) and TNFα (rho = 0.605, *p* < 0.001) pancreatic expression. Furthermore, CD45 intestinal expression was positively correlated with TNFα pancreatic expression (rho = 0.554, *p* < 0.001).

In the AP + eugenol group, HMGB1 intestinal expression and serum IL-6 were positively correlated (rho = 0.632, *p* < 0.001), while both were negatively correlated with TNFα (serum IL-6: rho = −0.643; HMGB1: rho = −0.573, *p* < 0.001) and MPO intestinal expression (serum IL-6: rho = −0.726; HMGB1: rho = −0.664, *p* < 0.001). A very strong positive correlation was evident between MPO and TNFα intestinal expression (rho = 0.906, *p* < 0.001).

## 4. Discussion

Earlier studies have reported the protective effect of eugenol following biliopancreatic duct ligation-induced AP on pancreatic and renal function [53,56]. According to Sowjanya et al., eugenol administration following AP induction reduced lipid peroxidation and enhanced cellular antioxidant defense mechanisms, thus preventing pancreatic and lung tissue damage [54]. In the present study, we investigated the anti-inflammatory effect of eugenol on rat intestinal tissue in an experimental model of AP. The rodent experimental model of biliopancreatic duct ligation-induced AP results in mild disease and multiple organ failure [58,59]. Relevant studies have reported increased TNFα and IL-6 levels in the serum, pancreas, and intestine, as well as increased pancreatic and intestinal MPO activity and altered gut homeostasis [33,34,35].

Histopathological changes in the intestinal tissue after AP induction were positively correlated with MPO, CD45, HMGB1, and TNFα intestinal expression, as well as IL-6 and TNFα pancreatic expression. The protective effect of eugenol in the intestinal tissue following AP induction was negatively correlated with IL-6 pancreatic expression, serum IL-6, and HMGB1, while it was also positively correlated with MPO and TNFα intestinal expression.

The induction of IL-6 takes place early in the onset of AP. Studies have reported increased plasma levels in patients with severe AP, 5 h and up to 48 h after admission, and a positive correlation with disease severity (renal, respiratory, and circulatory failure) [60,61,62]. TNFα plasma levels were positively correlated with renal, respiratory, hepatic, and circulatory failure in AP patients [62]. In the present study, we observed an increase in IL-6 and TNFα serum levels in the AP group. Eugenol treatment reduced IL-6 serum levels 48 h after the induction of AP in a time-dependent manner, but it did not affect TNFα serum levels. Eugenol (10.7 mg/kg) reduced TNFα and IL-6 plasma levels 48 h after the initiation of thioacetamide-induced liver injury in rats [63]. IL-6 pancreatic expression fluctuated within the first 72 h and reached a maximum at 48 h after the initiation of AP. Eugenol sustained IL-6 expression at similar levels from 12 up to 72 h and attenuated the upregulation of IL-6 at 48 h in the pancreas, while it caused a moderate increase in IL-6 expression at 72 h in the intestine. Similar results were observed in the kidney as well [56]. Serum IL-6 levels were also induced by eugenol at 6 h. Further studies are required to elucidate these observations. A direct or indirect mechanism implicating HMGB1, TNFα, and MPO seems probable. AP induced TNFα expression in the pancreas at 72 h. As in the kidney [56], AP induced TNFα intestinal expression 24 h earlier than in the pancreas, while eugenol exhibited an anti-inflammatory effect at 72 h in all three tissues. It should be noted that early in the onset of AP (6 h), IL-6 and TNFα pancreatic expression was downregulated in the AP group. In pancreatic acinar cells, CD45 and TNFα expression are negatively correlated [29]. It would be interesting to investigate how eugenol affects CD45 expression in pancreatic acinar cells.

HMGB1, in response to inflammatory stimuli, translocates from the nucleus to the cytosol, where it plays a role in regulating autophagy. It is also secreted to the extracellular space by activated tissue macrophages and monocytes, where it plays a primary role in the inflammatory response [64]. It is believed that DAMPs trigger the inflammatory response in AP [4]. Duct ligation-induced AP caused an early induction of HMGB1 in the intestinal tissue, which peaked at 24 h and remained at similar levels up to 72 h. It was mainly detected in the cytoplasm of epithelial cells, and it was positively correlated with MPO expression. Zhang et al. reported an increase in HMGB1 expression in the pancreatic tissue 12 h after the induction of necrotizing AP in rats that, similarly to our findings, maximized at 24 h [40]. Kang et al., reported that the knockout of pancreatic HMGB1 expression was associated with increased tissue damage and lethality in L-arginine- or cerulein-induced AP in mice. This study demonstrated that the intracellular release of HMGB1 in pancreatic acinar cells protects cells against DNA damage and cell death, while the extracellular release of HMGB1 from innate immune cells amplifies inflammatory responses and tissue injury [65]. Increased HMGB1 mRNA levels were reported in the ileum in an experimental model of acute necrotizing pancreatitis, while treatment with an HMGB1 neutralizing antibody decreased serum IL-1β, IL-6, and TNF-a serum levels and protected against intestinal mucosal barrier dysfunction [66]. HMGB1 administration affected the integrity of the intestinal barrier function by inducing iNOS and ONOO- formation in vitro and in vivo [67]. Additionally, increased serum HMGB1 levels in patients with severe AP were positively correlated with dysregulation in the intestinal barrier function [68]. Eugenol attenuated HMGB1 overexpression in the intestine 24 h after duct ligation-induced AP, which was positively correlated with serum IL-6 levels and negatively correlated with MPO and TNFα intestinal expression. Further studies are required to delineate if and how IL-6 and TNFα expression in the pancreas affects HMGB1 intestinal expression. Based on our results, the direct/indirect regulation of TNFα’s intestinal expression is a probable mechanism, but it is also highly probable that other factors are also involved.

CD45 expression was downregulated in pancreatic acinar cells 6 h after the initiation of AP by biliopancreatic duct ligation [29,35]. However, according to our findings, at the same timepoint, there was an increased population of CD45-expressing cells in the intestinal tissue, which could be attributed to inflammatory cell infiltration. In fact, CD45 expression was upregulated in macrophages present in the interstitial connective tissue, but it was also detected in the intestinal epithelium. Eugenol treatment effectively reduced CD45 expression. MPO-immunopositive cells increased 48 h after AP induction. MPO is considered a marker of phagocytic leukocyte migration, particularly of neutrophils, to sites of insult [69]. Unlike CD45, MPO is not expressed in tissue-resident macrophages, and it is weakly expressed in monocytes [70,71]. Based on our findings, the activation of intestinal-resident macrophages and intestinal CD45 upregulation takes place very early in duct ligation-induced AP. Furthermore, there is likely an association with TNFα’s pancreatic expression. According to our knowledge, there are no previous reports concerning the expression of CD45 in intestinal epithelial cells and, moreover, its upregulation in AP.

A strong connection between TNFα intestinal expression and phagocytic leukocyte migration was also evident, especially in relation to eugenol treatment. Overall, eugenol reduced the population of MPO-expressing cells from 12 h up to 72 h. MPO-expressing cells were significantly reduced in the pancreas after eugenol treatment at 48 h and 72 h in AP-induced rats, but there was no similar effect on the kidney [53,56]. Eugenol (10 mg/kg/day) also reduced MPO hepatic activity in a rat model of hepatic ischemia/reperfusion [72]. MPO is mainly expressed in neutrophils [30,31]. Eugenol attenuated neutrophil recruitment more effectively than the recruitment of other inflammatory cells by regulating the intestinal expression of TNFα, probably via its effect on the pancreatic expression of IL-6 and TNFα.

IL-18 plasma levels were positively correlated with renal and respiratory failure in AP patients. No correlation with intestinal, hepatic, or circulatory failure was evident [62]. We also did not observe any correlation between IL-18 serum levels and intestinal tissue lesions. Nevertheless, a transient increase was evident at 6 h, independently of eugenol treatment. A transient IL-18 upregulation was noticed within the first days after the onset of symptoms in patients with mild AP [73,74]. Eugenol induced IL-18 levels at 72 h as well. Corsini et al. reported that, at a concentration of 300 μg/mL, eugenol induced IL-18 release in human keratinocyte cell line NCTC 2544 [75]. In the present study, serum resistin levels were not correlated with TNFα or tissue lesions, as was observed in cerulein- and L-arginine-induced AP in rats [22]. Eugenol reduced resistin levels at certain timepoints after the initiation of AP. Further studies are required to delineate these observations.

In previous studies, eugenol attenuated *S.* Typhimurium-induced intestinal inflammation and tissue lesions by downregulating the expression of TNFα, IL-1β, IL-2, and IL-18, among others [76]. Moreover, it reduced TNFα, TNFβ, and INFγ expression in arthritic mice [77] and serum TNFα, IL-1β, IL-6, and NFκβ levels in a spinal cord injury experimental model in rats [78]. According to our knowledge, there are no reports on the effect of eugenol on resistin, CD45, and HMGB1 expression, in general. Moreover, there are no previous studies on the effect of eugenol on intestinal tissue damage caused by AP.

A limitation of our study could be that the half-life of eugenol in rats has been determined to be 18.3 h [79], and during our 72 h protocol, the initial dose would have been cleared from animals’ circulation. Subsequently, observations at additional timepoints could identify additional therapeutic properties in eugenol.

## 5. Conclusions

To conclude, eugenol managed to attenuate the inflammatory response in the intestine in duct ligation-induced AP in rats. TNFα, IL-6, HMGB1, CD45, and MPO seem to be, either directly or indirectly, implicated. Further studies are required to elucidate the underlying mechanisms. Moreover, it is worth performing similar studies on other AP experimental models.

## Figures and Tables

**Table 1:** Morphological changes in rat intestinal tissue after biliopancreatic duct ligation-induced pancreatitis (AP) with or without eugenol treatment. Scoring is presented as median and interquartile range.

		Hyperemia	Inflammatory Infiltrate in Mucosal Layer	Inflammatory Infiltrate in Submucosal Layer	Inflammatory Infiltrate in Muscular Layer
sham	6	1 (0.25–1)	0.50 (0–1)	0.5 (0–1)	0 (0)
	12	0.50 (0–1)	0 (0)	0 (0)	0.5 (0–1)
	24	1 (0.25–1)	0 (0)	0 (0)	0 (0–0.75)
	48	0.50 (0–1)	0 (0–0.75)	0 (0)	0 (0)
	72	1 (0.25–1)	0 (0–0.75)	0 (0)	0 (0)
AP	6	0 (0–1)	0 (0)	0 (0)	0 (0)
	12	1 (0.25–1)	0 (0)	0 (0)	0 (0–1)
	24	1 (1)	0 (0–0.75)	0.5 (0–1)	0 (0)
	48	1.50 (1–2) *	0.5 (0–1)	1 (0.25–1)	0 (0–1)
	72	2 (2) *	1 (1) *	1 (1) *	1 (1) *
AP + eugenol	6	0.50 (0–1)	0 (0)	0 (0)	0 (0)
	12	0 (0–1)	0 (0)	0 (0)	0 (0)
	24	0 (0–1) **	0 (0)	0 (0)	0 (0)
	48	1 (1)	0 (0–0.75)	0 (0–0.75)	1 (0–1)
	72	1 (1–1.75) **	0.50 (0–1) **	1 (0–1) **	1 (0–1) **

* *p* < 0.05 vs. the corresponding sham subgroup, ** *p* < 0.05 vs. the corresponding AP subgroup.

## Data Availability

Data are online available.
